# Distributed Fiber Optics Sensing and Coda Wave Interferometry Techniques for Damage Monitoring in Concrete Structures

**DOI:** 10.3390/s19020356

**Published:** 2019-01-16

**Authors:** Antoine Bassil, Xin Wang, Xavier Chapeleau, Ernst Niederleithinger, Odile Abraham, Dominique Leduc

**Affiliations:** 1 IFSTTAR, COSYS-SII, Route de Bouaye, F-44344 Bouguenais, France; xavier.chapeleau@ifsttar.fr; 2 Inria, Team I4S, Campus de Beaulieu, F-35042 Rennes, France; 3 Bundesanstalt für Materialforschung und-prüfung (BAM), Unter den Eichen 87, 12205 Berlin, Germany; xin.wang@bam.de (X.W.); ernst.niederleithinger@bam.de (E.N.); 4 IFSTTAR, GERS-GeoEND, Route de Bouaye, F-44344 Bouguenais, France; odile.abraham@ifsttar.fr; 5 GeM UMR 6183, University of Nantes, F-44322 Nantes, France; dominique.leduc@univ-nantes.fr

**Keywords:** distributed fiber optic sensors, coda wave interferometry, reinforced concrete, cracks, damage detection, structural health monitoring

## Abstract

The assessment of Coda Wave Interferometry (CWI) and Distributed Fiber Optics Sensing (DFOS) techniques for the detection of damages in a laboratory size reinforced concrete beam is presented in this paper. The sensitivity of these two novel techniques to micro cracks is discussed and compared to standard traditional sensors. Moreover, the capacity of a DFOS technique to localize cracks and quantify crack openings is also assessed. The results show that the implementation of CWI and DFOS techniques allow the detection of early subtle changes in reinforced concrete structures until crack formation. With their ability to quantify the crack opening, following early detection and localization, DFOS techniques can achieve more effective monitoring of reinforced concrete structures. Contrary to discrete sensors, CWI and DFOS techniques cover larger areas and thus provide more efficient infrastructures asset management and maintenance operations throughout the lifetime of the structure.

## 1. Introduction

The continuous growth in worldwide population and the climate changes (affecting the probability of natural hazards) are increasing the need for housing and better infrastructures. Nowadays, reinforced concrete is the most employed material in the construction industry, but the global trend is to reduce its consumption rate and thus to change the focus from “design of new structures” to “maintenance of the current constructions” [1]. For this reason, Structural Health monitoring (SHM) systems will play an increasingly important role. The main idea of SHM is to compare the “as-is” structural condition, which includes the damage, fatigue, load distribution, etc., to the “as-built” structural condition, which comes from the structural design. Then, the models can be updated so that the structural integrity can be evaluated based on the “as-is” model [2]. Civil infrastructure monitoring is required in cases where structures are subject to long-term degradation of materials like fatigue and where a feedback loop is needed to improve future design based on experience (like in the case of bridges and wind turbine foundations). Currently, the majority of research activities in the SHM area are focused on developing sensing technologies and damage detection algorithms [3]. Sensors developed within other engineering disciplines, such as Distributed Fiber Optics Sensing (DFOS) and Coda Wave Interferometry (CWI) techniques, are now finding their way into civil applications. Implementing an autonomous SHM system supporting structural maintenance depends on dealing with some key problems like data storage, environmental effects, communications, inappropriate instrumentation, and the lack of collaboration. In addition, careful selection of sensors and their locations are important to obtain useful information about the structural behavior. On the other hand, introducing multi-sensors based systems with different damage sensitivity ranges, that could be complementary in some ways, seems also paramount. Thus, there is a need for the development of comparative studies employing different techniques of structural assessment and damage detection [4,5].

Traditional strain sensors can either be discrete Strain Gauges (SG), which are usually attached to the surface, or vibrating wire sensors, which are usually embedded inside the structure. In addition, Linear Variable Differential Transformers (LVDTs) are also used for strain monitoring. With a limited number of these point sensors, the global behavior of a structure can be monitored. However, it will be hard to follow any unexpected and unusual localized damage. In order to overcome this limitation, DFOS or CWI techniques can be used. DFO sensors installed over the length of the structure can provide spatially distributed strain measurements and therefore direct damage detection and localization can be achieved [6]. On the other hand, CWI can detect subtle changes in heterogeneous materials like concrete [7,8]. This allows large structures to be monitored using a limited number of sensors. However, the superiority of novel sensors over traditional sensors needs to be proved by studying their reliability and discussing their advantages in real-life situations.

The aim of this paper is to show the added value of DFOS and CWI dual instrumentation for structural health monitoring compared to usual instrumentation with classical SG and LVDT sensors. The two techniques are first presented, then the experiment on a laboratory size reinforced concrete beam is described and, finally, the results are discussed.

## 2. Distributed Fiber Optics Sensing (DFOS) Technique

### 2.1. Working Principle

DFOS techniques can be compared to having a large number of sensors regularly spaced along an optical fiber and thus, providing distributed measurements over a large section of the structure. Brillouin and Rayleigh backscattering based techniques [6] are the two strain sensing techniques available today in the market. While the first one is the result of interaction between photons and phonons causing frequency shifts (proportional to strain), the second one is caused by the sub-wavelength variations in the fiber’s index of refraction. These variations form a sort of fingerprint for every optical fiber. Thus, when a light beam is injected inside the optical fiber, part of this light beam is backscattered, and when compared to a reference signal, a frequency shift can be calculated. This frequency shift is proportional to the change in strain and temperature.

Former distributed sensing techniques, based on Rayleigh power loss measurement (Optical Time Domain Reflectometry OTDR) [9,10] or Brillouin frequency shift (Brillouin Optical Time Domain Reflectometry BOTDR and Brillouin Optical Time Domain Analysis BOTDA) [11,12,13,14], have low spatial resolutions (10 cm to 1 m), which are insufficient for direct crack detection and for quantification of the crack opening. With the recent developments of new Rayleigh based DFOS techniques like Tunable Wavelength Coherent Optical Time Domain Reflectometry TW-COTDR [15,16] or Optical Frequency Domain Reflectometry OFDR [17], more accurate strain measurements with millimeters spatial resolutions can be obtained. Although this may form an asset for localized damage detection in reinforced concrete structures, these techniques are limited to small range distances compared to Brillouin based techniques capable of reaching 1–100 km range.

### 2.2. From Strain to Crack Monitoring

**Figure 1:**
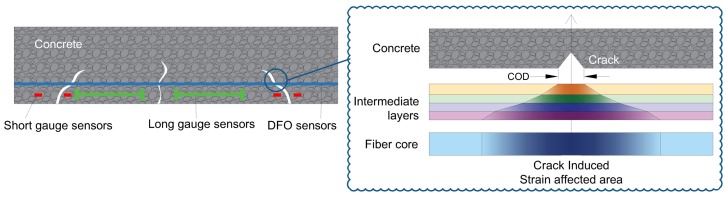
Crack detection using Distributed Fiber Optics Sensing (DFOS) techniques.

As mentioned in the previous paragraph, DFOS techniques provide spatially distributed strain measurements. Thus, as shown in Figure 1, direct damage detection and localization can be achieved contrary to short gauge and long gauge sensors where sophisticated algorithms are required [18]. Previous works [19,20,21,22,23] have already demonstrated the ability of DOFS to detect and localize cracks in concrete structures. However, the quantification of crack opening assessment from this kind of measurements remains a challenge that this paper aims to tackle. For civil structures applications, optical fibers are usually surrounded by protective layers or adhesives in order to prevent fiber breakage and to glue the fiber on the surface of the structure [24]. As shown in Figure 1, it was found that the deformation discontinuity due to the crack formation is transferred to the optical fiber, through the intermediate layers, and in the form of a localized strain distribution covering an important length (several centimeters) of the optical cable [22].

In [25], Feng et al. proposed a mechanical model based on shear lag theory in order to explain this phenomenon. By assuming a linear elastic behavior of the different constitutive layers of the cable and a perfect bonding at the different interfaces [26], the strain measured by the optical fiber $\epsilon_{fiber}\left(x\right)$ near the crack location is equal to the sum of the strain in the host material $\epsilon_{concrete}\left(x\right)$ and the Crack Induced Strain (CIS):

(1) $$CIS=\frac{COD}{2}\;\beta e^{‒\beta |x‒c|},$$

where COD, *c* and β represent respectively the Crack Opening Displacement, the position of the crack and the shear lag parameter. This latter depends on the mechanical and geometrical properties of the cable. Low thickness of intermediate layers leads to high shear lag parameter values and therefore higher strain transfer. In like manner, higher stiffness of intermediate layers increases also the strain transfer.

Few efforts to apply this model for quantification of crack openings, either on steel [27], aluminum [23,28] or reinforced concrete structures [29], exist in the literature. However, the limitations of the used DFOS systems, in terms of spatial resolution and measurement rate, affected the accuracy of the results.

## 3. Coda Wave Interferometry (CWI) Technique

### 3.1. Working Principle

For SHM applications, the working frequency range for sonic methods or vibration measurement is normally below 10 kHz. As a result, the wavelength is larger than the size of many typical defects or aggregates. On the other hand, ultrasonic measurements are performed in a frequency ranges that exceed 50 kHz, forcing the waves to enter the multiple scattering regime and interact with small heterogeneities [30]. As diffused waves travel along much longer paths than direct or simply reflected ones, they are much more sensitive to weak perturbation in the medium. Henceforth, ultrasonic CWI is considered, nowadays, one of the most promising methods for detection of subtle changes in heterogeneous materials like concrete.

**Figure 2:**
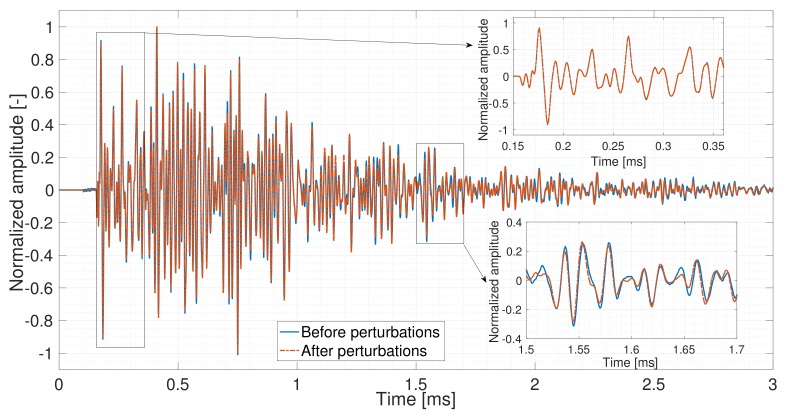
Signals recorded before and after perturbation in the medium [31].

The principle of CWI is to compare the coda waves recorded in two different states to monitor weak velocity variations and waveform modifications. As shown in Figure 2, two signals are recorded before and after a perturbation. The first arrivals of the signals are almost the same (i.e., [0.16 ms, 0.35 ms]), while the coda wave (i.e., [1.5 ms, 1.7 ms]) shows a significant difference. For this reason, CWI is more sensitive to weak perturbation in the medium.

The most used method to evaluate these changes is the stretching method [32] where the velocity change is considered as dilation or compression in time by a factor α. This method is based on choosing a reference signal $u_{u}\left(t\right)$ and then stretching it by different dilation rates α in the range $\left[\alpha_{min},\alpha_{max}\right]$. Cross correlation between the signal in a new state $u_{p}\left(t\right)$ and all the stretched reference signals $u_{u}\left(t\left(1+\alpha \right)\right)$ are then calculated. The basis of the CWI method is the correlation coefficient (CC) which measures the similarity of the signals and the velocity change (dV/V) within a certain time window [t‒T, t+T]:

(2) $$CC\left(\alpha \right)=\frac{\int_{t‒T}^{t+T}u_{u}\left(t^{{}^{\prime }}\left(1+\alpha \right)\right)u_{p}\left(t^{{}^{\prime }}\right)dt^{{}^{\prime }}}{\sqrt{\int_{t‒T}^{t+T}u_{u}^{2}\left(t^{{}^{\prime }}\left(1+\alpha \right)\right)dt^{{}^{\prime }}\int_{t‒T}^{t+T}u_{p}^{2}\left(t^{{}^{\prime }}\right)dt^{{}^{\prime }}}}.$$

The parameter α, which maximizes the cross correlation, is considered as the velocity change, while the CC variation indicates a local change (e.g., stress change or permanent local change as cracks). By comparing the coda waves measured in two different states, weak changes in the medium can be monitored and quantified.

### 3.2. Standard and Stepwise CWI Procedures

As a standard procedure, one or several fixed signals recorded before changes in the structure are chosen as reference signals. Cross correlation and velocity change are determined compared to this reference. However, when the changes in the structure exceed a certain limit (i.e., waveforms change completely or waveforms shifted by more than half of the wavelength), the velocity change is meaningless while the CC might still be useful as it measures the similarity of two signals. In this case, standard CWI procedure is not applicable anymore. One way to deal with this limit is to calculate stepwise changes by comparing $u_{p}^{n}\left(t\right)$ with the previous signal $u_{p}^{n‒1}\left(t\right)$ (stepwise CWI) as implemented in [33]. Since stepwise CC can only show the similarity between current and previous signals, the stepwise CWI should be combined with standard CWI for long-term monitoring in order to detect the occurrence of any unusual behavior. The main purpose of the test is to monitor the first preliminary crack. In this way, it was decided to stop the test after the creation of the second crack.

## 4. Experimental Investigation

### 4.1. Test Set-Up

**Figure 3:**
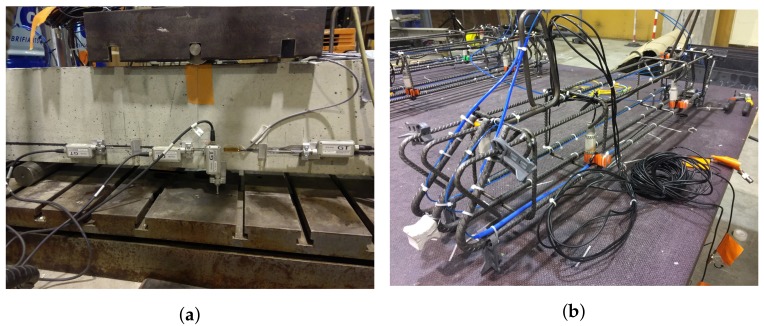
(**a**) front view of the loading arrangement and the beam instrumented with sensors before testing; (**b**) Ultra Sonics (US) and Distributed Fiber optics (DFO) sensors attached to the rebars before casting of concrete.

**Figure 4:**
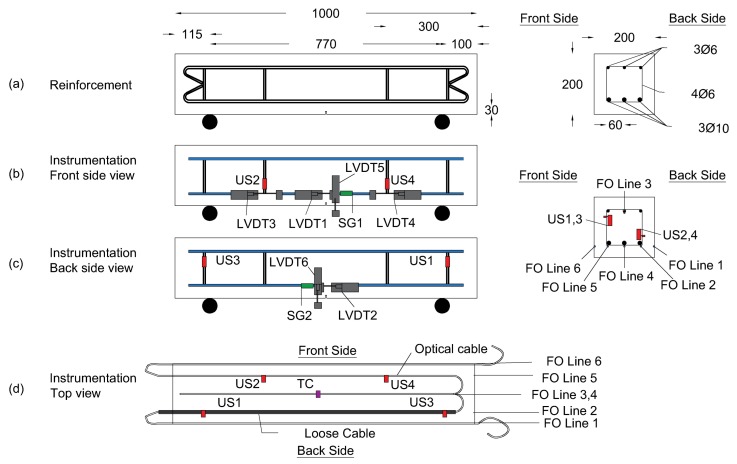
(**a**) dimensions of the beam and different rebars positions; (**b**) front view of sensors positions; (**c**) back view of sensors positions; (**d**) top view of sensors positions.

A 20 × 20 × 100 cm reinforced concrete beam was tested under continuous three-point loading with a loading speed of 1 kN/min (Figure 3a). As shown in Figure 4a, the beam was reinforced with three ϕ10 longitudinal reinforcement bars (rebars) in the tension area and three ϕ6 rebars in the compression area, attached together by four ϕ6 stirrups. The beam was instrumented with SG (green color), LVDT (grey color), Ultra Sonic US (red color) and DFO (blue color) sensors. Before casting of concrete, four US sensors were attached to the stirrups while one optical cable was fixed over the length of four rebars (Figure 3b). After casting, the same optical cable was bonded to the surface of the beam by making a groove in the concrete at the same level of rebars and gluing the optical cable using a two components epoxy adhesive.

Figure 4 shows the top view of the optical cable trajectory while respectively:

- Glued on the front surface (Line 1).
- Attached to the front bottom rebar (Line 2). This line was surrounded by a tube to create a loose part for temperature compensation.
- Attached to the mid top rebar (Line 3).
- Attached to the mid bottom rebar (Line 4).
- Attached to the back bottom rebar (Line 5).
- Glued on the back surface (Line 6).

In addition, two LVDT sensors (one from each side) were measuring deflection at the center of the beam, while four other sensors were fixed at the level of rebars for displacement monitoring at specific locations as shown in Figure 4b,c. Moreover, two SG sensors (one from each side) were fixed 6 cm away from the central part of the beam. A thermocouple (purple color), fixed in the central part of the beam, followed low temperature variations (in the order of ±0.1 ${}^{\circ }$C) during the test in accordance with those measured all over Line 2 (Figure 4d).

#### 4.1.1. DFO System Set-Up

**Figure 5:**
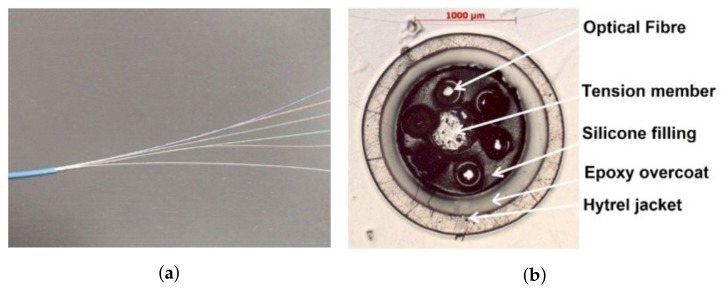
(**a**) Photo of the optical cable. (**b**) Micrography of different constitutive layers of the optical cable [22].

ODISI–B interrogator (manufactured by Luna, Blacksburg, VA, United States) was chosen for this experiment. Based on Optical Backscattering Reflectometry (OBR) technique, this interrogator can reach a spatial resolution of 5.2 mm with a maximum strain repeatability of ±10 μm/m and a strain accuracy of ±25 μm/m. Figure 5 shows the AFL optical cable (from Sensornet, Hertfordshire, United Kingdom) used as a sensor. As shown in Figure 5b, the cable holds six fibers wrapped around a central rod and embedded in a soft polymer matrix. One of the optical fibers was connected to the interrogator and used for performing the measurements.

#### 4.1.2. Ultrasonic System Set-Up

**Figure 6:**
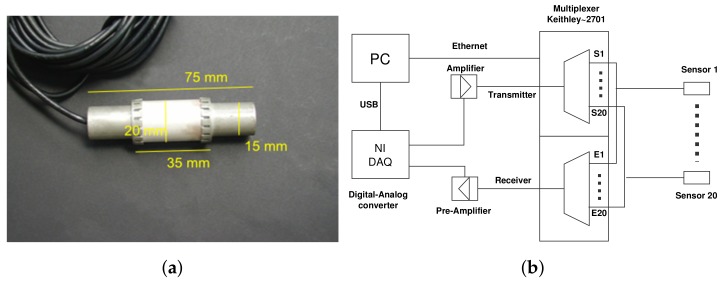
(**a**) dimensions of “SO807” [34]; (**b**) diagram of the data acquisition system.

A new embedded ultrasonic transducer “SO807” was used for this experiment. It was designed by Acoustic Control Systems ACS (Sarrebruck, Germany) in cooperation with and exclusively for BAM [34]. The main part of SO807 is a hollow piezo ceramic cylinder (Figure 6a) that can be both transmitter and receiver, and can be installed easily during the construction. The central frequency of this transducer is around 62 kHz. Contrary to classic ultrasonic sensors glued on the structure surface, SO807 is embedded inside the concrete material and thus records fewer surface waves and is less influenced by near-surface changes (e.g., temperature influence). As shown in Figure 6b, a Keithley 2701 multiplexer (manufactured by Linktronix, Thalwil, Switzerland) was used to switch between different combinations S-E (transmitter-receiver) during the test. This data acquisition system allows autonomous continuous monitoring of the structure with a sampling frequency of 1 MHz.

### 4.2. Test Results

#### 4.2.1. Deflection Measurements

**Figure 7:**
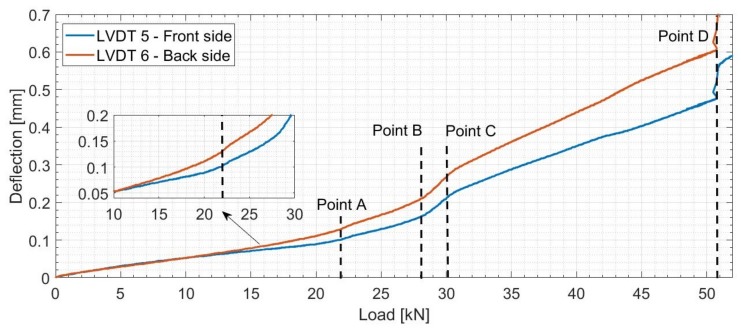
Variation of the vertical deflection in the central part of the beam measured by Linear Variable Differential Transformers (LVDT) sensors.

Back and front side vertical deflection values measured by LVDT sensors are plotted in Figure 7, in which the four marked points mark out each change in the curve shape and therefore a change in the behavior of the reinforced concrete beam.

Point A (load = 22 kN) marks the end of a linear elastic phase and the start of nonlinear behavior of the beam. This nonlinear behavior is due to the initiation of small micro cracks in the central part of the beam where the highest value of bending moment is located. Between Point B and Point C, the beam goes through a remarkable increase in deflection rate. Hence, this state marks a rapid reduction in the stiffness of the beam and therefore high deterioration problems. The jump in deflection observed when the load reached 51 kN (Point D) is believed to be due to the formation of a macro crack. Furthermore, distributed load and geometric imperfections of the beam could explain the difference in front and back side deflection values. This difference becomes more evident after the beam enters its nonlinear state.

#### 4.2.2. DFO Strain Measurements

**Figure 8:**
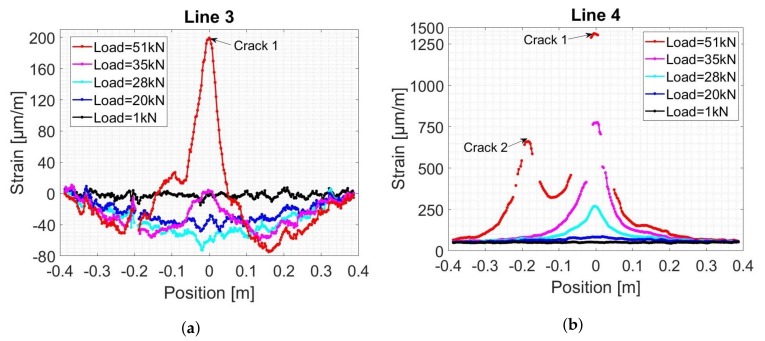
Spatial strain distribution in concrete measured by the DFOS system near the top (**a**) and bottom (**b**) rebars.

The DFO strain measurements were performed with a frequency rate of 10 Hz and then the moving average over ten consecutive measurements was calculated. The spatial strain distribution over the length of FO Line 3 (near the top rebars) and Line 4 (near the bottom rebars) at five different load levels is plotted in Figure 8. Fixed at the level of the bottom rebars, Line 4 detects a micro crack when the load level increases from 20 kN to 28 kN. The strain profile evolves exponentially, indicating that a first crack, located near the central part of the beam, reaches the level of the bottom rebars. For regular concrete, the strain in the tension part does not exceed 100μm/m. With the increase in the crack opening, strain reigning in concrete material becomes therefore negligible when compared to the CIS that extends spatially to more than 40 cm of the length of the cable. On the other hand, FO Line 3 fixed at the top rebars shows the negative triangular strain distribution in the compression part of the beam. Strain profile at 35 kN load level shows higher values in its central part. The exponential shape indicates that the same crack propagates over the height of the beam until reaching top rebars. When the load reaches 51 kN, strain distribution in Line 4 shows another exponential increase indicating that a second micro crack appears around 20 cm from the center of the beam. While COD value increases, and due to rapid strain transitions near the crack location, a high number of dropouts are filtered by ODISI-B interrogator software (Version 5.2.2).

#### 4.2.3. CWI Velocity Change (dV/V) and Correlation Coefficient (CC) Measurements

**Figure 9:**
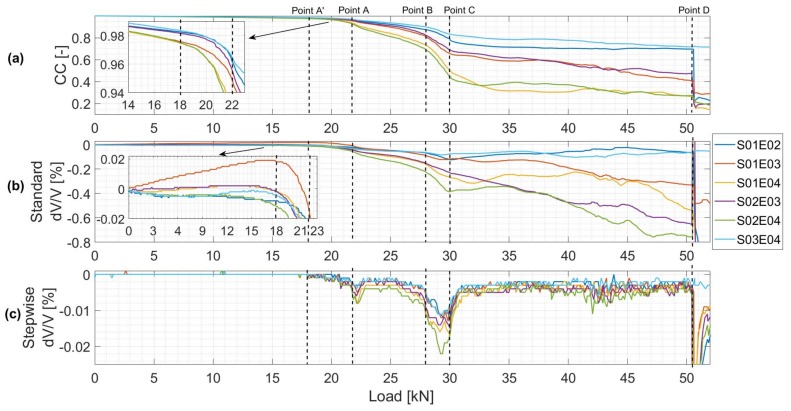
(**a**) CC plots for each SxxEyy combination. (**b**) dV/V plots for each SxxEyy combination. (**c**) Stepwise dV/V plots for each SxxEyy combination.

The CC and dV/V values, calculated by standard and stepwise CWI for all different combinations of sensors SxxEyy (transmitter xx and receiver yy), are plotted in Figure 9. A reference signal was chosen for standard CWI calculations before any load was applied. The CC values for all different combinations decrease as the load increases continuously during the test (Figure 9a). CC and deflection plots took a similar trend as they are related to the stiffness of the reinforced concrete beam. As micro cracks propagate over the height of the beam, the similarity between the ultrasonic signals reduces and is accompanied by a rapid decrease in CC values. Similarly, standard dV/V values decrease during the test depending on stress changes and positions of the sensors (Figure 9b). During the linear elastic state, these values vary between 0.02% and −0.01% for S01E03 and S02E04 combinations where the two sensors were positioned, respectively, in the upper and bottom part of the beam. While dV/V decreases for combination S02E04, S01E03 dV/V increases linearly with the increase in compression stresses in the upper part of the beam. Indeed, the stress–velocity variation coefficient changes between different combinations depending on the location and the distance between the two transducers [34]. Local velocity changes compared with the previous signal are observed clearly from stepwise dV/V (Figure 9c). The changes in stepwise dV/V plots help distinguishing four main remarkable load levels similar to those observed in the deflection plots. CWI results and their relation to damage propagation will be discussed and compared with other sensors in Section 5.1.3.

## 5. Discussion

After presenting the test results in Section 4.2, early damage detection and sensitivity of DFOS and CWI techniques to damage propagation are evaluated in this section and compared to other standard sensors. Then, the possibility of estimating the COD using the mechanical transfer function to DFO strain profiles are demonstrated.

### 5.1. Damage Detection

#### 5.1.1. DFOS Technique

The damage detection properties of DFOS technique can be defined by evaluating the changes in strain profiles due to a crack formation. These changes are dependent on the cables strain transfer mechanism and the interrogator properties. In terms of strain transfer (described in Equation (1)), higher shear lag parameter values would induce higher sensitivity to micro crack propagation for the DFO system used in this study. As mentioned in [25], the shear lag parameter is related to mechanical and geometrical properties of the different intermediate layers between the optical fiber and the host material. Talking about the interrogator properties, and due to the exponential shape of CIS distribution, higher spatial resolution means better sensitivity to strain variations between two sampling points and therefore better sensitivity to micro cracks. In addition, better accuracy of strain measurements and its repeatability can improve the detection capacity of the system.

**Figure 10:**
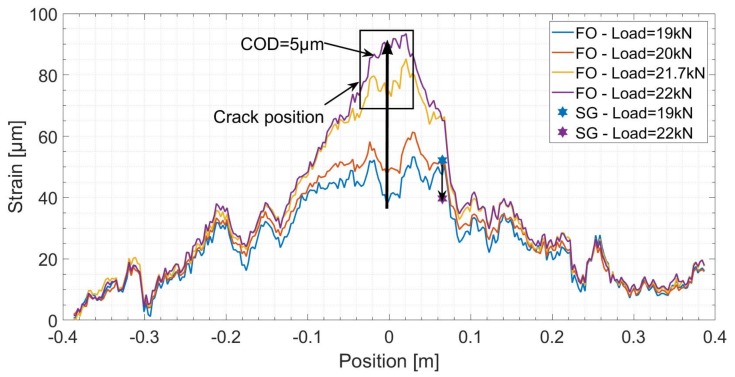
Spatial strain distribution over the length of Fiber Optics (FO) Line 1 before and after the creation of the first micro crack.

Figure 10 shows the measured strain versus the position for different load levels. For the specific (cable, glue, concrete) combination used in this experimentation, a crack opening of 5μm resulted in an increase to 200% in the strain measured near the location of the crack. On the other hand, the strain measured by SG sensors decreased to 80% at 6 cm far from the crack location. If the crack location were closer to the SG sensors, a bigger rate of strain decrease would have been detected. However, if the crack bridged through the SG sensor, high strain values would have been measured. Since these two cases are rare when instrumenting real scale structures, crack detection using SG sensors is in most cases an indirect detection.

At the crack location (x=c), Equation (1) can be written as follows:

(3) $$\begin{array}{c} CIS=\frac{\beta }{2}\;COD. \end{array}$$

The shear lag parameter, estimated by fitting Equation (1) to the measured strain profiles, has a constant value that varies between 22 and 28 m${}^{‒1}$ for each FO Line. Thus, the DFO system with a strain resolution (1 μm/m) and strain repeatability (±2 μm/m) can therefore detect the change in CIS around 5–7 times less than $\frac{\beta }{2}$ value. While the latter corresponds to an increase of 1μm in COD, LVDT sensors used in this experiment with a resolution of 1μm, are therefore 5–7 times less sensitive to micro crack propagation than the DFO system.

#### 5.1.2. CWI Technique

CC is related to the changes in the media. The more the media changes, the less is the value of CC. Mechanical stress can induce changes in the elastic wave velocity [35]. A previous experiment on the acousto-elastic effect (relation between stress and acoustic velocity) showed that weak velocity change under weak load variation is almost linear [36]. By observing a slope change in CC and dV/V, creation of a small crack can be detected. In addition, CC and dV/V properties of the US signal can express the severity of cracking incidences. As a result, two different types of crack propagation can be distinguished:

- Accumulated micro cracks: micro cracks are developed under minute stresses. Increasing stress can connect these micro cracks and lead to the creation of cracks which remain permanent and are not reversible [35].
- Brittle macro crack formation: the formation of these cracks is accompanied by an important amount of internal energy release and therefore velocity change losses that can exceed 1% [35].

#### 5.1.3. Comparison

**Figure 11:**
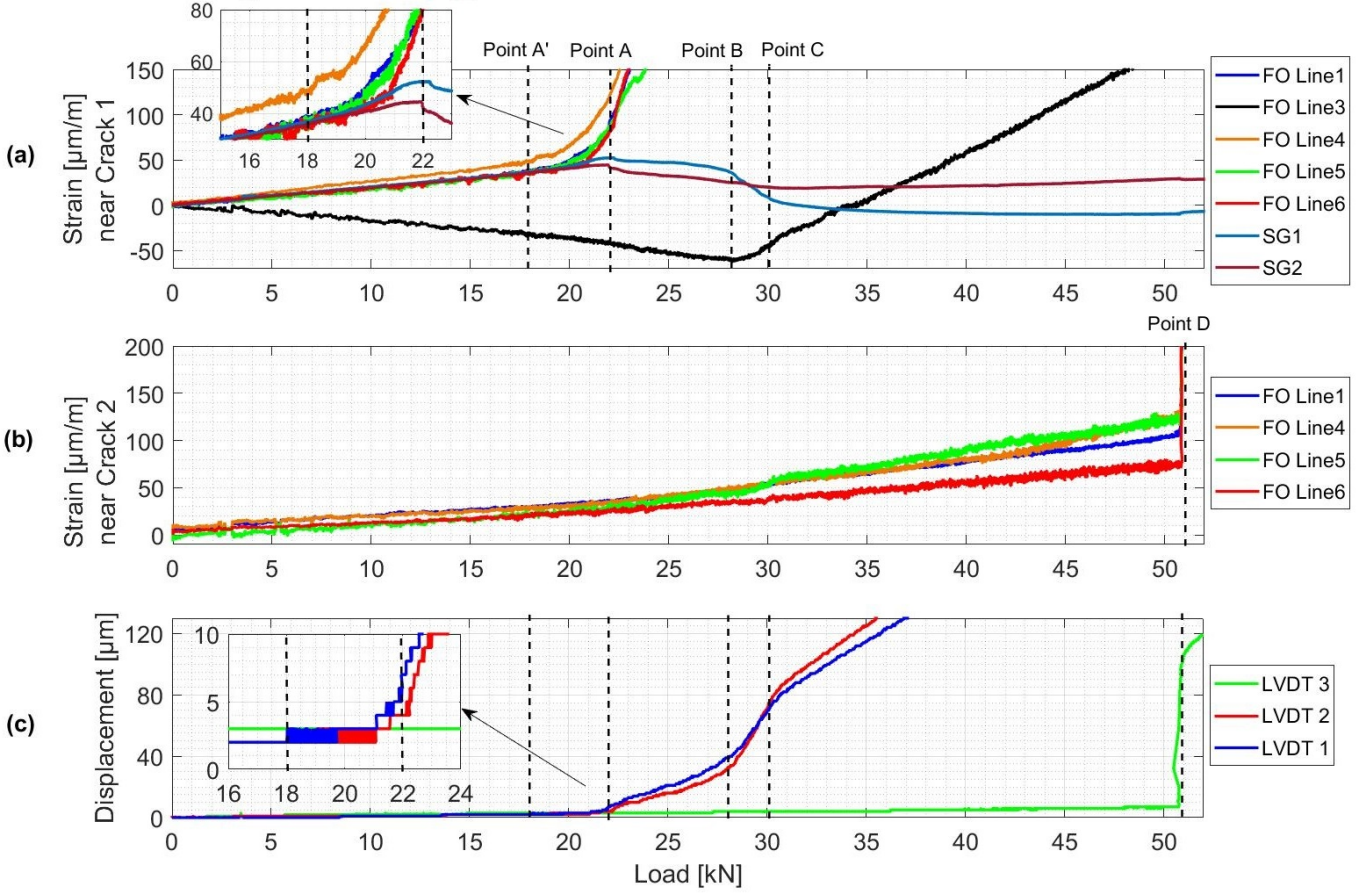
(**a**) Strain variations measured by FO lines and SG sensors near Crack 1. (**b**) Strain variations measured by FO lines near Crack 2. (**c**) Horizontal displacements measured by LVDT sensors.

While Figure 9 presents the results of CC and dV/V calculated from US sensors measurements, Figure 11 shows the DFO strain measurements at the location of cracks 1 and 2 compared to displacements (crack openings) and strains measured respectively by LVDT (1, 2, 3) and SG (1, 2). Cracks 1 and 2 refer to the first crack detected at the center of the beam and the second one 20 cm away from the center (monitored by LVDT 3).

Similar to the previous observations from deflection plots (Figure 7), four different points can be differed:

- Point A’: formation of Crack 1 in the center of the beam.
- Between Point B and C: Crack 1 reaching the top rebars level.
- Point D: formation of Crack 2 at 20 cm from the center of the beam.

##### Point A’: Formation of a First Crack

The formation of crack 1 is first detected by US and DFO sensors. When the load exceeded 18 kN, a first variation in velocity for S02E04 combination is observed from stepwise dV/V plot (Figure 9c). This decrease in dV/V followed shortly by other combinations is due to the propagation of the micro crack. The standard dV/V plots show that this fall in dV/V value is the highest between the two closest transducers located on the bottom part of the beam (combination S02E04). On the other hand, the smallest variations are observed for combinations S01E02 and S03E04 as the direct waves between these transducers do not bridge through the crack. CC plots follow a similar trend as dV/V plots.

Likewise, FO Line 1 and 4 present first an increase in strain variation near 18 kN, followed shortly by Line 5 and 6 near 20 kN (Figure 11a). However, the change in LVDT 1 and LVDT 2 measurements due to the crack opening occurred when the load reached 21 kN with higher values on the front side of the beam (Figure 11c). The creation of Crack 1 was the reason behind a strain release in concrete. This decrease in strain was detected by SG (1, 2) 6 cm from the crack location near 22 kN (Figure 11a). Finally, LVDT 3 did not show any change in measured displacement.

##### Points B–C: The First Crack Reaching the Top Rebars

At about 28 kN, strain measured by FO Line 3 (attached to the top rebars) started increasing and therefore indicated the detection of Crack 1. A stable propagation of Crack 1 over the height of the beam, due to a load increase, is noticed from the linear decrease in dV/V and CC values between 18 kN and 28 kN. When Crack 1 approaches top rebars, a high dV/V and CC changing rates are observed signaling a rapid crack propagation between the top rebars level and the top of the beam until the load reached 30 kN. The beam is then divided into two parts and therefore dV/V values started increasing for combinations S01E02 and S03E04 (Figure 9b). The fact that the top rebars started working in tension near the crack introduced a sort of asymmetrical damage propagation in the beam. This can be observed from LVDT (1, 2) and SG (1, 2) measurement plots where the CODs and strains measured on the back side of the beam reached higher values than those on the front side between 28 kN and 31 kN (Figure 11c).

##### Point D: Formation of a Second Crack

When load reached 51kN, Crack 2 suddenly appeared at 20 cm from the center of the beam. The fact that the crack reached instantaneously a COD of 100μm led to high variations in strain measured by FO Line (1, 4, 5, 6) (Figure 11b). For US measurement, the CC and dV/V for all combinations, except S03E04, changed significantly (Figure 9). Indeed, transducers (03, 04) are located on the other side of the beam and direct waves do not pass through the second crack. Another piece of compelling evidence is that the beam was separated into two parts.

### 5.2. Estimation of the Crack Opening Displacement

**Figure 12:**
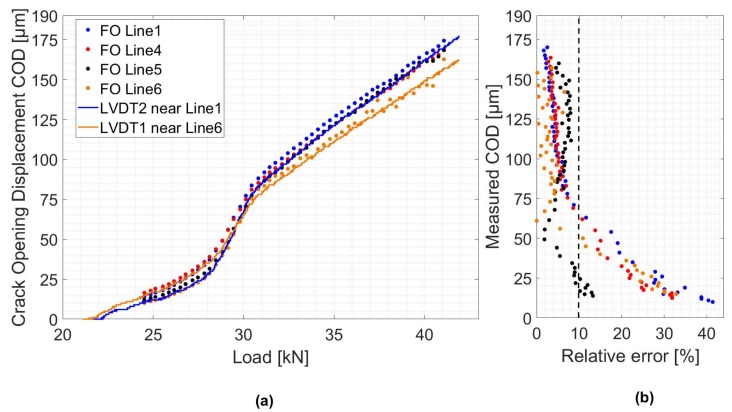
(**a**) Estimated crack openings compared to LVDT measurements. (**b**) Relative error compared to LVDT measurements.

As mentioned in Section 5.1.1, the CIS distribution (Equation 1) was fitted to the measured strain profiles using the least square method. δ and β were selected as variable parameters. Figure 12a shows a comparison between the estimated CODs from different FO Lines and those measured with LVDT sensors. The measured CODs near FO lines 4 and 5 are first determined by assuming a linear variation between the front and back sides values measured by LVDT 1 and 2. Then, the absolute relative error values for each FO Line are calculated (Figure 12b). For all different FO Lines, a relative error of less than 10% is achieved when the COD exceeds 65μm. While a better accuracy is reached with the increase in the COD, the number of dropout points increases. As a result, and due to a lack of measurement values near the crack location, the fit does not converge for CODs exceeding 175μm.

## 6. Conclusions and Outlook

In this work, CWI and DFO new sensing techniques were combined. Their capacity to detect early damage in reinforced concrete structures was evaluated and compared to other standard traditional sensors like strain gauges and LVDT displacement sensors measuring deflection and crack openings. CWI and DFO sensors achieved prior damage detection than standard sensors without being dependent on the location of the structural defect. The combination of these two NDT techniques allows for explaining different types of change in the behavior of the reinforced concrete beam.

The estimated crack openings, compared to LVDT sensors working as crack meters, showed a small relative error in the order of 10%. On a real structure like a bridge, a crack map can be established and crack openings can be continuously monitored. However, in order to use a DFOS technique for crack opening monitoring, special laboratory experiments dedicated to single crack propagation case should be performed in order to study the shear lag parameter variations and search for the suitable cable configuration for concrete applications.

For CWI techniques, a relationship between the crack depth and velocity change and correlation coefficient should be established and then generalized for multiple cracks case. Even though the CWI method has shown great sensitivity to the detection of stress change and cracking, the position of cracks can only be roughly inferred. As a final step, imaging of stress distribution and crack localization can be established.

For field applications, and especially for reinforced concrete structures exposed to cyclic loads during their lifetime, the degradation of these sensors should be studied by performing fatigue tests. In addition, the issue of cross sensitivity to temperature (for DFO and CWI techniques), moisture and various damage mechanisms should be addressed. As a result, the implementation of these sensors in a structural health monitoring system will help in understanding long-term phenomenon like fatigue by studying in depth the nature of the crack deterioration and help in decision-making.

## Abbreviations

The following abbreviations are used in this manuscript:

BAM	Bundesanstalt für Materialforschung und-prüfung
CC	Correlation Coefficient
CIS	Crack Induced Strain
COD	Crack Optical Displacement
CWI	Coda Wave Interferometry
DFOS	Distributed Fiber Optics Sensing
FBG	Fiber Bragg Grating
FO	Fiber Optics
FOS	Fiber Optics Sensors
IFSTTAR	The French Institute of Science and Technology for Transport, Development and Networks
LVDT	Linear Variable Differential Transformer
NDT	Non-Destructive Testing
OBR	Optical Backscattering Reflectometry
SG	Strain Gauge
SHM	Structural Health Monitoring
US	Ultra Sonics
